# Safety and Efficacy of Salt Restriction Across the Spectrum of Heart Failure

**DOI:** 10.3390/jcdd12110432

**Published:** 2025-11-02

**Authors:** Panagiotis Stachteas, Athina Nasoufidou, Markella Koiliari, Vasiliki Arampatzi, Chrysa Alexaki, Christos Kofos, Paschalis Karakasis, Efstratios Karagiannidis, Theocharis Koufakis, Nikolaos Fragakis, Dimitrios Patoulias

**Affiliations:** 1 Second Department of Cardiology, Aristotle University of Thessaloniki, Hippokration General Hospital of Thessaloniki, Konstantinoupoleos 49, 54642 Thessaloniki, Greece; athinanassi@gmail.com (A.N.); markellako79@gmail.com (M.K.); vikismil84@gmail.com (V.A.); xrisalex5@gmail.com (C.A.); chriskofos21@gmail.com (C.K.); pkarakaa@auth.gr (P.K.); ekaragij@auth.gr (E.K.); nfrag@auth.gr (N.F.); 2 Second Propedeutic Department of Internal Medicine, Aristotle University of Thessaloniki, Hippokration General Hospital of Thessaloniki, Konstantinoupoleos 49, 54642 Thessaloniki, Greece; thkoyfak@auth.gr (T.K.); patoulias@auth.gr (D.P.)

**Keywords:** heart failure, salt restriction, sodium intake, non-pharmacological strategies

## Abstract

Dietary sodium restriction is widely recommended in heart failure (HF) management; however, its benefits and risks remain a subject of ongoing debate. While moderate sodium reduction may improve symptoms and quality of life in selected patients, excessive restriction can trigger maladaptive neurohormonal activation, worsen renal function, and increase the risk of hyponatremia, malnutrition, and cachexia. Patient response is heterogeneous, influenced by clinical risk profile, salt sensitivity, comorbidities, and age, with some high-risk patients experiencing neutral or adverse outcomes. Additional challenges arise from hidden sodium in processed foods, medications, and meals, which complicate monitoring and adherence. Effective sodium management in HF therefore requires a nuanced, individualized approach that integrates risk stratification, dietary counseling, and public health measures targeting the food industry. Future research should refine patient selection criteria and establish optimal sodium targets to balance therapeutic efficacy with safety in real-world practice.

## 1. Introduction

Heart failure (HF) remains a major global health burden, affecting over 64 million individuals worldwide and associated with significant morbidity, mortality, and healthcare costs [1,2]. Despite substantial therapeutic advancements in recent years—particularly with the advent of neurohormonal antagonists, sodium glucose co-transporter 2 (SGLT2) inhibitors, and device therapies [3]—the syndrome continues to pose clinical challenges due to its complex pathophysiology and progressive nature [4].

In parallel with pharmacologic innovation, increasing attention has been directed toward non-pharmacological strategies that may complement medical therapy and improve outcomes. Among these, dietary sodium restriction has long been advocated as a cornerstone of HF management, with the rationale that reduced sodium intake may limit fluid retention and alleviate congestion, a cardinal symptom of the disease [5,6,7].

However, the evidence supporting sodium restriction in HF remains surprisingly inconclusive and sometimes contradictory [7]. While international guidelines continue to recommend dietary sodium restriction, often without specifying an optimal target or acknowledging differences across the HF spectrum [2,8,9], recent randomized trials have challenged the universality of this recommendation. Moreover, the impact of sodium restriction on significant clinical endpoints such as hospitalizations, quality of life, and mortality is still under debate, and concerns have been raised about potential adverse effects, particularly in more advanced stages of the disease.

This review aims to critically appraise the current evidence on the safety and efficacy of dietary sodium restriction across the spectrum of HF phenotypes, highlight gaps in evidence, and discuss implications for clinical practice and future research.

## 2. Pathophysiological Basis of Sodium Restriction in Heart Failure

### 2.1. Sodium and Volume Overload

**Figure 1:**
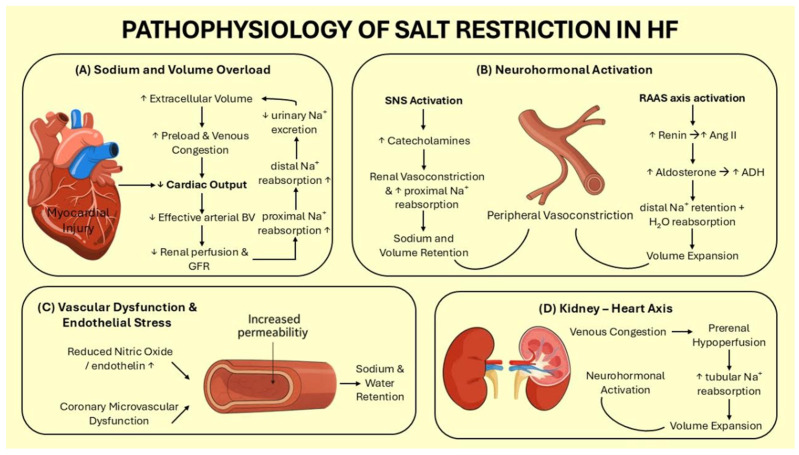
The pathophysiologic basis of sodium restriction in heart failure progression.

Sodium retention in HF results from a wide array of components that act synergistically to preserve perfusion in the setting of reduced cardiac output. In the highly complex pathophysiological cascade of HF, the initiating event is structural damage of the myocardium, presenting either as a physical loss of myocardial cells or as a functional impairment [10]. Shortly after myocardial dysfunction has been established, various compensatory mechanisms—metabolic, neurohormonal, and structural—are activated in an attempt to restore functional equilibrium with left ventricular remodelling being the most prominent amongst them (Figure 1A) [10]. Over time, these mechanisms eventually fail and become maladaptive further contributing to HF progression.

Normally, only 30–40% of total blood volume is in the arterial circulation and this proportion is even lower in systolic HF. To maintain tissue perfusion, significant systemic volume expansion occurs as a compensatory response to preserve effective circulating volume and cardiac function [11]. Over time, this mechanism becomes maladaptive, leading to excessive blood volume, fluid retention, and organ congestion, characterized by elevated central venous and cardiac filling pressures [12]. The extent of this retention is influenced by the buffering capacity of the interstitial compartment. Normally, the interstitial compartment can hold 3–4 times more fluid than the intravascular space [13]. In steady-state conditions, a balanced interplay between transcapillary hydrostatic and oncotic pressures—commonly described by Starling’s principle—ensures the absence of net fluid movement across the capillary wall. In the context of HF, however, impaired systolic function leads to reduced cardiac output (CO). In HF, reduced CO triggers a compensatory shift in fluid from the interstitial space into the vasculature, increasing plasma volume to help restore circulating volume and maintain organ perfusion [14].

### 2.2. Neurohormonal Activation

In early HF, reduced CO activates the sympathetic nervous system (SNS) and suppresses the parasympathetic system through baroreceptor and mechanoreceptor signals. This leads to β1-receptor downregulation, β2- and α1-receptor upregulation, and elevated norepinephrine, causing tachycardia and vasoconstriction. Renal hypoperfusion stimulates renin release, driving sodium and water retention via the renin–angiotensin–aldosterone system (RAAS) [15].

Renin, secreted by the juxtaglomerular apparatus, converts angiotensinogen to angiotensin I, which ACE then converts to angiotensin II. Acting via G protein-coupled receptors, angiotensin II causes vasoconstriction, apoptosis, thirst, and pro-inflammatory and pro-thrombotic responses, while partly exerting anti-inflammatory effects through type II receptors. Its main role is stimulating aldosterone release, which increases sodium reabsorption and potassium excretion in the distal tubule, raising blood volume and preload [16]. Chronic RAAS activation, however, promotes interstitial fibrosis in the heart, kidneys, and vessels, while paradoxically reducing effective circulating volume, perpetuating SNS and RAAS activation [17].

Angiotensin II also triggers antidiuretic hormone (ADH) release. ADH’s V1a receptors increase vascular resistance and contribute to cardiac remodelling, while V2 receptors in renal collecting ducts promote water retention by inserting aquaporin-2 channels [18]. This enhances free water reabsorption, often causing dilutional hyponatremia in advanced HF [19].

In response to sodium and water retention, the myocardium releases natriuretic peptides. Atrial natriuretic peptide (ANP), secreted mainly from the right atrium during dilation, counters neurohormonal activation via G-protein–coupled receptors [20]. ANP promotes sodium and water excretion, reduces sympathetic activity, inhibits renin and aldosterone, lowers vascular resistance, and limits cardiac hypertrophy and fibrosis. Similarly, B-type natriuretic peptide (BNP), released from the ventricles under chronic stress, has comparable effects. Its precursor, NT-proBNP, is clinically favoured for HF diagnosis due to its longer half-life [21]. Despite opposing SNS and RAAS activation, natriuretic peptides alone cannot fully restore homeostasis (Figure 1B).

### 2.3. Vascular Dysfunction & Endothelial Stress

In HF, beyond fluid and sodium shifts, endothelial dysfunction significantly contributes to disease progression. This dysfunction disrupts vascular tone, thrombosis regulation, and systemic balance [22]. Normally, nitric oxide (NO) promotes vasodilation via cGMP signaling, but in HF, oxidative stress and reduced NO synthesis impair vasodilation, leading to poor skeletal muscle perfusion and exercise intolerance (Figure 1C).

Coronary microvascular dysfunction further limits myocardial perfusion due to endothelial abnormalities. Inflammation, particularly TNF-α activation, fosters fibrosis and inhibits NO synthase, worsening vasomotor control and repair mechanisms [23]. Elevated angiotensin II, norepinephrine, and hypoxia increase endothelin-1 production, causing vascular smooth muscle contraction, hypertrophy, proliferation, and ultimately greater vascular stiffness and reduced arterial compliance.

### 2.4. Kidney–Heart Axis

Cardiorenal syndrome describes the bidirectional dysfunction of the heart and kidneys, where impairment in one organ worsens the other and complicates HF management due to reduced GFR [24]. Traditionally, it is viewed as heart-centered: reduced CO causes arterial underperfusion, increased atrial pressure, and venous congestion [25]. This prerenal hypoperfusion activates neurohormonal responses (SNS, RAAS, vasopressin, endothelin-1), promoting vasoconstriction and sodium/water retention. While these mechanisms temporarily preserve blood pressure and perfusion, they raise afterload, lower CO, and worsen renal function (Figure 1D). Elevated blood urea, due to increased tubular reabsorption, indirectly indicates neurohormonal activation [26].

Earlier theories linked reduced CO directly to lower GFR, but the ESCAPE study showed that improving cardiac index does not always enhance renal function [27]. Other factors, including elevated intra-abdominal or central venous pressure and right ventricular dilation, also impair GFR by raising renal venous pressure and reducing left ventricular preload. Additionally, endothelial dysfunction and inflammatory cytokines (TNF-α, IL-1, IL-6) contribute to cardiorenal syndrome, as they exert cardiodepressant effects and lower left ventricular ejection fraction (EF) [28].

## 3. Historical Perspectives and Guideline Evolution

Dietary sodium restriction has long been a cornerstone of HF management, with traditional targets of <2 g/day tracing back to the pre-diuretic era, when limiting salt was one of the few available strategies to control edema and congestion. These recommendations were based more on pathophysiological reasoning—and, at times, extrapolated from arterial hypertension guidelines—than on evidence from blinded clinical trials [29]. The notable variation among major guidelines reflects the limited strength of the supporting data. Importantly, the evidence base should be considered separately for chronic and acute HF, as outcomes and therapeutic implications may differ between these populations [30].

European Society of Cardiology (ESC) have gradually downgraded recommendations about sodium intake over time, now advising the avoidance of excessive salt intake (<5 g/day) [31]—although sodium and salt are often used as synonyms, they are not exactly the same; salt is made of 40% sodium and 60% chloride [32]—, in the current 2021 guidelines for the management of HF, emphasizing individualized counseling rather than universal limits due to the lack of evidence for stricter restrictions [33]. The 2022 American Heart Association/American College of Cardiology/Heart Failure Society of America guidelines advise sodium intake below 2.3 g/day for general cardiovascular health, and in stage C HF, suggest avoiding excessive intake to reduce congestion while acknowledging the absence of robust trial evidence in this population [34]. The Canadian Cardiovascular Society suggests a target of dietary sodium intake of 2–3 g/day [35], while the World Health Organization recommends <2 g/day for general cardiovascular disease prevention in adults [36]. Consequently, the existing clinical evidence remains inconsistent, resulting in no clear consensus among major current HF management guidelines regarding the optimal level of dietary sodium restriction.

## 4. Clinical Evidence Across the Spectrum of Heart Failure

A narrative literature review of the existing evidence was undertaken to synthesize and critically appraise the heterogeneous data on the safety and efficacy of dietary sodium restriction across the spectrum of HF. The PubMed and Google Scholar databases were systematically searched up to May 2025 using Boolean operators (AND, OR, NOT) with appropriate combinations of the following search terms: “heart failure,” “salt restriction,” “sodium restriction,” “dietary sodium,” and “randomized controlled trial.” Reference lists of eligible studies were also screened to identify additional relevant publications. Only articles written in English were included, while gray literature, conference abstracts, and non-peer-reviewed sources were excluded. From the initial pool of retrieved records, duplicates were removed, and a first-level screening was performed based on titles and abstracts. Full-text articles were subsequently assessed for eligibility, and studies directly comparing different levels of dietary sodium intake in adult patients with HF were included. Ultimately, 17 randomized controlled trials (RCTs) met the inclusion criteria and are summarized in Table 1. Fifteen studies employed a parallel-group design, while the remaining two followed a cross-over design. The majority of the RCTs (*n* = 13) recruited predominantly outpatients, mainly individuals living in the community and diagnosed with chronic HF. In contrast, four studies examined hospitalized patients with ADHF, representing a distinct clinical profile in terms of severity and management setting.

Regarding the specific HF phenotypes, nine RCTs exclusively included patients with HF with reduced ejection fraction (HFrEF). Only one trial included patients with HF with preserved ejection fraction (HFpEF), while the remaining studies enrolled mixed populations, including individuals with HFrEF and/or HFpEF. The underlying etiology of HF varied considerably; however, ischemic origin was predominant in most cohorts. The burden of comorbid conditions also demonstrated significant variability between trials. Specifically, the prevalence of hypertension ranged from 28.0% to 98.4%, diabetes mellitus from 31.6% to 56.0%, and ischemic heart disease from 18.2% to 76.6%, reflecting the clinical heterogeneity of the included populations.

The nature and extent of the dietary sodium restriction varied notably between studies. Eleven RCTs implemented a strict sodium intake restriction of less than 2 g/day, while in the remaining six, the restriction was more moderate, ranging between 2 and 3 g/day. Furthermore, in six studies, fluid intake was also restricted to less than 1500 mL/day in the intervention group. In some cases, fluid restriction was applied equally to both the intervention and control groups, making the assessment of sodium-specific effects more complex.

The primary and secondary outcomes reported across studies included all-cause mortality, hospital readmissions, changes in NYHA functional class, and quality of life. It is important to note that six RCTs did not report mortality outcomes, which limits the robustness of mortality-based conclusions. Also, nine RCTs did not provide any hospitalization data, further complicating comparative analysis. Regarding functional status, as assessed by the NYHA classification, ten studies did not report any relevant findings. Of the remaining trials, three found no significant improvement in NYHA class, while the others documented notable improvement. This shift in functional status was particularly evident in the trial conducted by Colin-Ramirez et al., highlighting the potential benefit of sodium restriction in symptomatic improvement [44].

Among the 17 RCTs summarized in Table 1, only a subset evaluated patient-reported quality of life (QoL) outcomes, predominantly using validated instruments such as the Minnesota Living with Heart Failure Questionnaire (MLHFQ) and the Kansas City Cardiomyopathy Questionnaire (KCCQ). Seven trials did not assess or report QoL results. Of those that did, four reported no significant difference between groups, while three demonstrated measurable improvements with moderate sodium restriction. Specifically, observed enhanced scores in the physical limitation and symptom frequency domains, suggesting better functional capacity and reduced congestion in the intervention groups [38,42]. Colin-Ramirez et al. also documented significant improvement in overall QoL scores, consistent with alleviation of dyspnea and fatigue [47]. Conversely, Philipson et al. (2010, 2013) and Colin-Ramirez et al. (2015) found no appreciable changes in QoL despite similar sodium restriction targets, possibly reflecting short intervention duration or concomitant fluid restriction [43,45,47]. Notably, the study by Kostis et al. reported a decline in QoL within the control arm, implying that the absence of structured dietary guidance may negatively affect patient perception of well-being [38]. Collectively, these findings indicate that moderate sodium restriction may improve physical and symptomatic domains of QoL, likely through reduced volume overload and improved exercise tolerance, although results remain inconsistent across trials.

Evidence from chronic HF trials has been mixed. The SODIUM-HF trial found that targeting < 1.5 gr/day sodium did not reduce clinical events in patients with chronic HF [53]. Recent meta-analyses have similarly shown no benefit for sodium restriction on HF-related hospitalizations or mortality, with improvements limited to symptoms and QoL [54], and some analyses have even suggested potential harm, with worsened composite outcomes including mortality and hospitalization [55]. In acute decompensated HF (ADHF), the evidence is even more complex. A meta-analysis found that sodium supplementation combined with furosemide improved renal function, reduced brain natriuretic peptide levels, promoted greater weight loss, and shortened hospital stays compared with furosemide alone [56]. An observational study in diuretic-resistant ADHF patients reported that hypertonic saline improved serum electrolytes, creatinine, urine output, weight loss, and diuretic efficiency, without adverse respiratory or neurological effects [57].

In conclusion, dietary sodium restriction in patients with HF shows potential benefits in terms of functional status and quality of life, particularly among patients with HFrEF. However, the lack of high-certainty evidence and inconsistent outcome reporting underscore the need for large-scale, well-designed, and adequately powered RCTs to better define the role and optimal level of sodium restriction in HF management. Until such data are available, clinical recommendations should be made with caution, considering individual patient characteristics, tolerability, and risk of adverse outcomes such as hyponatremia or reduced intake of essential nutrients [58].

## 5. Risks and Controversies

### 5.1. Excessive Sodium Restriction Risks

While sodium restriction has traditionally been advocated for patients with HF, emerging evidence raises concerns about its potential adverse effects, particularly when implemented aggressively. Randomized data have shown that combining low dietary sodium intake (<2 g/day) with diuretic therapy can activate counter-regulatory mechanisms such as the RAAS -resulting in increased renin secretion by the kidneys and consequently aldosterone- and SNS [59,60]. This neurohormonal activation may increase cardiac workload, elevate left ventricular filling pressures, and worsen myocardial oxygen demand—factors detrimental to HF prognosis [61]. Additionally, strict sodium restriction has been associated with a higher risk of hyponatremia, which is itself linked to prolonged hospitalizations, increased readmission rates, and worse overall outcomes [59,61,62].

Further concerns relate to nutritional and renal complications. Studies combining sodium and fluid restriction with high-dose diuretics have reported rises in serum creatinine and urea, suggesting an increased risk of renal impairment due to intravascular volume depletion [60,63]. In elderly HF patients, sodium restriction has also been linked to reduced appetite, lower caloric intake, and malnutrition—often exacerbated by pre-existing taste disturbances from reduced salivation, zinc deficiency and polypharmacy [64]. These nutritional deficits may promote cachexia and sarcopenia, conditions associated with frailty and poorer clinical outcomes [65].

Excessive dietary sodium restriction may influence not only cardiovascular and renal physiology but also systemic osmolar homeostasis. Alterations in extracellular osmolarity have been shown to induce a range of metabolic and cellular effects. Experimental studies indicate that exposure to a hyperosmotic extracellular milieu leads to cellular shrinkage and activation of catabolic pathways in several tissues, including the liver, skeletal muscle, and skin [66]. These adaptive mechanisms promote proteolysis, gluconeogenesis, and amino acid release, potentially resulting in protein-energy wasting and insulin resistance. In patients with HF—particularly those with advanced disease or cachexia—such osmotic and metabolic disturbances may further exacerbate frailty and adversely affect clinical outcomes [66]. Taken together, these findings underscore the need for a cautious, individualized approach to sodium restriction in HF patients, balancing potential hemodynamic benefits against the risks of neurohormonal activation, renal dysfunction, malnutrition, maladaptive osmotic stress and metabolic deterioration [64].

### 5.2. Heterogeneity of Response

The response to dietary sodium restriction in HF is not uniform across all patients, with growing evidence highlighting significant heterogeneity in both efficacy and safety. Data from the SODIUM-HF study and subsequent post hoc indicate that patients at low risk of mortality, as defined by the MAGGIC HF risk score, may derive clinical benefit from sodium restriction—particularly when quality-of-life measures such as the Kansas City Cardiomyopathy Questionnaire (KCCQ) score are favorable. Conversely, in high-risk patients, sodium restriction appears less effective and may even be detrimental, reinforcing the need for risk-stratified dietary interventions [67].

This variability may, in part, be explained by salt sensitivity, a condition traditionally associated with blood pressure (BP) variability but now recognized as a broader cardiovascular risk factor—even in normotensive individuals [68,69]. Salt sensitivity is more prevalent in older adults, women, individuals of African descent, and those with CKD or insulin resistance. Underlying mechanisms include impaired renal sodium handling (due to defective natriuresis), endothelial dysfunction (via reduced NO availability), and heightened neurohormonal activation involving the RAAS and SNS [68,69]. These pathophysiological differences underscore the importance of identifying salt-sensitive individuals and tailoring sodium intake recommendations accordingly, rather than adopting a blanket restriction strategy in all HF patients.

### 5.3. Challenges in Monitoring Sodium Intake

The accurate quantification of sodium intake remains a major methodological challenge both in HF research and in daily clinical practice. Although multiple 24 h urinary sodium excretions are considered the gold standard for estimating intake, their application in HF populations is limited. Diuretic therapy, incomplete urine collection, and dynamic changes in fluid balance may substantially distort sodium excretion data [29]. Consequently, most trials have relied on dietary recalls, weighed food records, or spot urine sampling to estimate sodium consumption, each method carrying varying degrees of bias and imprecision. These methodological differences likely contribute to the heterogeneity of findings across studies evaluating the efficacy and safety of sodium restriction in HF.

Despite the clinical rationale for sodium restriction in HF, practical implementation remains challenging due to hidden sources of sodium and lifestyle-related factors [70]. Processed and low-fat foods, often marketed as healthy options, frequently contain high levels of sodium to enhance flavor, while store-brand products have been shown to contain significantly more sodium than branded equivalents [71]. Additionally, over-the-counter medications such as paracetamol and ibuprofen can contribute substantial hidden sodium—up to 3 gr/day when taken at standard doses—posing a serious, often overlooked risk in HF patients [72]. These issues are compounded by limited patient awareness and inconsistent labeling practices by the food industry. As such, effective sodium reduction requires not only patient education and behavioral change, but also broader policy and industry reforms to ensure transparency and facilitate healthier choices [70,71,72]. Merely advising patients to avoid table salt [32] is insufficient without comprehensive strategies that address these systemic and environmental contributors to excess sodium intake.

## 6. Public Health and Nutritional Considerations

### 6.1. Population-Wide Sodium Reduction

Although population-level sodium reduction lowers BP and cardiovascular risk, its role in HF management remains nuanced. Excess dietary sodium intake (>5 g/day) is strongly associated with hypertension and related cardiovascular complications [73,74]. Numerous studies and meta-analyses confirm that reducing sodium -even with interventions such as salt substitutes—lowers BP (both systolic and diastolic), improves general cardiovascular health and decreases cardiovascular morbidity and mortality [75,76,77].

However, evidence of population-wide sodium and salt reduction in HF patients is limited and the current data are quite heterogenous. Some studies suggest that strict sodium restriction may increase mortality and hospitalizations [78], especially in advanced HF or in those on high-dose diuretics, possibly due to volume depletion or electrolyte imbalances. Conversely, mild salt restriction combined with fluid control may offer benefits in selected patients [78,79]. For instance, among HF patients with NYHA III/IV symptoms, intake > 3 g/day was linked to worse outcomes for cardiac event-free survival, whereas in milder HF (NYHA I/II), it appeared protective [80]. Other studies showed neutral results of sodium restriction on deaths or hospitalizations in patients with HF. For instance, a RCT trial investigating the effects of a 2 g/day sodium restriction versus a 3 g/day control in stable HFrEF patients found no significant improvements in cardiac parameters or quality of life [52].

These conflicting results suggest that while sodium restriction may offer benefits in certain HF populations, it may not be universally advantageous highlighting the need for personalized dietary strategies in HF, based on clinical status, comorbidities, and medication use, rather than a one-size-fits-all approach.

### 6.2. Caloric Adequacy, Protein Intake, and Micronutrient Needs

A comprehensive dietary strategy is essential in the holistic management of HF, particularly among elderly or frail patients who are at increased risk for malnutrition. While sodium and fluid restriction remain a cornerstone of dietary guidance, it must be carefully balanced against the broader nutritional demands of this population—including adequate caloric, protein, and micronutrient intake—to avoid exacerbating frailty or clinical deterioration [81,82].

Undernutrition is prevalent in HF and contributes to adverse outcomes such as muscle wasting, immunosuppression, and increased hospitalization risk [81]. Despite recommendations for protein intake of ≥1 g/kg/day to preserve lean mass, many patients fail to meet these targets [83]. Micronutrient deficiencies, notably in calcium, magnesium, and vitamins D and E, are also common and clinically relevant [81,84].

Elderly or frail individuals face additional barriers to adequate nutrition, including anorexia, dysphagia, dental issues, and multimorbidity, which complicate dietary adherence [81,85]. These factors heighten the risk for both under- and over-nutrition, underscoring the need for individualized dietary planning that considers functional limitations, cultural context, and meal preparation capacity. Therefore, nutritional interventions in HF should go beyond sodium and fluid reduction and adopt a patient-centered, holistic approach aimed at maintaining nutritional adequacy and improving outcomes [85].

### 6.3. Importance of Dietary Counseling, Shared Decision-Making and Cardio-Nutrition Teams

Diet is a pivotal modifiable factor in CVD prevention and management, and its relevance is particularly pronounced in HF. Patient-centered strategies that include structured dietary counseling, shared decision-making (SDM), and the integration of cardio-nutrition teams are increasingly recognized as essential to improving clinical outcomes and adherence to lifestyle interventions [86,87]. Dietary patterns such as the Mediterranean and DASH diets are consistently associated with reductions in hypertension, ischemic events, and HF incidence. However, translating these broad recommendations into individualized, sustainable plans in HF management often requires high-quality counseling by registered dietitians. Evidence supports the efficacy of intensive dietary interventions in improving cardiometabolic parameters—including BP, lipid profiles, and glycemic control—as demonstrated by recent meta-analyses [86,87].

SDM—a collaborative process where clinicians and patients work together to make informed health decisions—further enhances these interventions by aligning dietary and therapeutic choices with patient values, improving engagement, adherence and reducing decisional conflict in chronic disease management [88]. Recognizing its impact, the American Heart Association advocates for SDM as a standard of care in cardiovascular medicine, particularly in areas such as lifestyle modifications and nutrition-related decisions [89].

The interdisciplinary cardio-nutrition model, comprising cardiologists, dietitians, behavioral experts, and allied health professionals, offers coordinated and patient-tailored care. This approach facilitates consistent messaging, supports sustained behavior change, and may reduce care fragmentation and readmissions [90]. Despite its potential, widespread adoption faces systemic barriers, including limited reimbursement for nutrition services, time constraints in primary care, unequal access to specialized teams, and lack of standardized SDM tools [91,92]. Future efforts should focus on scalable digital tools for dietary guidance, expanded reimbursement models for interdisciplinary services, and rigorous evaluation of the long-term clinical and economic impact of cardio-nutrition teams. Integrating these approaches within value-based care frameworks holds promise for reducing CVD burden and enhancing patient-centered outcomes in HF and beyond [91,92].

## 7. Future Directions

Despite decades of clinical guidance advocating for sodium restriction in HF management, the evidence remains inconsistent, largely due to heterogeneity in patient populations and trial designs [53]. There is a clear need for more pragmatic RCTs that reflect real-world settings and patient diversity. Future studies should stratify patients by EF, as the pathophysiological mechanisms and responses to dietary interventions may differ significantly between those with preserved, mildly reduced, and reduced EFs. Additionally, baseline sodium intake must be considered as a variable, as individuals with already low sodium consumption may respond differently to further restriction compared to those with high intake. Stratification by salt sensitivity and comorbidities such as CKD, hypertension, or diabetes is also critical, given that these conditions can modulate both the risks and benefits of sodium restriction [85]. By embracing these stratification strategies, future RCTs can generate more nuanced and clinically applicable data, ultimately guiding personalized dietary recommendations for HF patients.

Personalized nutrition strategies represent a promising avenue for optimizing HF care. Biomarkers of salt sensitivity may enable targeted sodium restriction, aligning with precision medicine approaches and improving individual responses to dietary interventions [69]. Concurrently, AI-driven tools and wearable technologies could facilitate real-time sodium tracking and feedback, supporting adaptive, patient-specific dietary adjustments [93]. Telehealth and remote coaching further enhance adherence through ongoing education and behavioral support, potentially improving long-term outcomes and enhancing patient engagement.

Integrating structured nutrition services into HF management programs remains a critical but underutilized opportunity. Multidisciplinary models involving dietitians, cardiologists, and allied health professionals can address not only sodium intake, but also energy balance, hydration, physical activity and comorbidity management [94,95]. Embedding dietary counseling into standard care and cardiac rehabilitation may improve adherence, reduce readmissions, and support long-term lifestyle modification [96,97]. Future research should prioritize scalable, tech-enabled interventions and reimbursement models that support interdisciplinary, nutrition-focused care.

## 8. Conclusions

In summary, while moderate sodium reduction can benefit selected patients with HF, excessive or indiscriminate restriction may activate maladaptive neurohormonal pathways, worsen renal function, and contribute to electrolyte disturbances and malnutrition. The heterogeneity of patient response—driven by factors such as clinical risk profile and salt sensitivity—underscores the need for individualized dietary targets. Effective implementation further requires addressing hidden sodium sources, improving food labeling, and enhancing patient education. Ultimately, a nuanced, patient-centered approach is essential to balance the benefits and risks of sodium restriction in HF management.

## Figures and Tables

**Table 1:** Randomized controlled trials investigating the effects of dietary sodium restriction in patients with heart failure.

Study ID	Population/Method of Sodium Assessment	Setting/ HF Type	Sample Size	Intervention/ (Duration)	Comparator	Outcome (MACE)
Cody et al., 1986 [37]	Hospitalized patients with moderate to severe chronic HF/24 h urinary sodium excretion	Inpatient/ HFrEF	10	Very low sodium restriction (230 mg/d)/ (14 days)	Low sodium intake (2300 mg/d)	NA
Kostis et al., 1994 [38]	Patients with chronic HF and NYHA II–III/Dietary recall and counseling logs	Outpatient/ HFrEF	13	Sodium restriction (1200 mg/d)/ (84 days)	Routine dietary advisories	Improvement in QoL and mood indices
Colin-Ramirez et al., 2004 [39]	Patients with HF based on reduced systolic and diastolic function in echo/3-day food records analyzed by dietitian	Outpatient/ HFrEF	65	Sodium restriction at 2000–2400 mg/d/ (180 days)	Routine dietary advisories	NS difference in NYHA class, significant improvement in QoL
Alvelos et al., 2004 [40]	Patients with mild to moderate chronic HF/24 h urinary sodium excretion	Outpatient/ HFrEF	24	Sodium restriction (2300 mg/d)/ (15 days)	Diet with usual salt intake	NS difference in NYHA class
Damgaard et al., 2006 [41]	Male patients with ADHF/24 h urinary sodium excretion	Outpatient/ HFrEF	12	Sodium restriction (1610 mg/d)/ (7 days)	High sodium intake (5750 mg/d)	NA
Nakasato et al., 2010 [42]	Patients with chronic HF and NYHA II–III/Dietitian-recorded menus with compliance interviews	Outpatient/ HFrEF	50	Very low sodium restriction (800 mg/d)/ (7 days)	Low sodium intake (2400 mg/d)	Improvement in QoL
Philipson et al., 2010 [43]	Patients with chronic HF and NYHA II–IV and signs of fluid retention/3 consecutive 24 h urine collections	Outpatient/ HFrEF or HFpEF	30	Sodium restriction at 2000–3000 mg/d and fluid restriction/ (84 days)	Routine dietary advisories	NS difference in QoL
Colin-Ramirez et al., 2010 [44]	Patients with HF based on reduced systolic and diastolic function in echo/3-day food records; subset urinary sodium validation	Outpatient/ HFrEF or HFpEF	203	Sodium restriction at 2000–2400 mg/d and fluid restriction/ (1 year)	Routine dietary advisories	Significant reduction in CV hospitalization
Philipson et al., 2013 [45]	Patients with chronic HF and NYHA II–IV and signs of fluid retention and maximal tolerated doses of GDMT/Dietary records and repeated spot urinary sodium	Outpatient/ HFrEF or HFpEF	97	Sodium restriction at 2000–3000 mg/d and fluid restriction/ (84 days)	Routine dietary advisories	NS difference in mortality, NYHA class or QoL
Aliti et al., 2013 [46]	Hospitalized patients with ADHF and LVEF < 45%/Controlled inpatient meal sodium records	Inpatient/ HFrEF	75	Very low sodium restriction at 800 mg/d and fluid restriction/ (7 days)	Diet with usual salt and fluid intake	30-day readmission rate: NS
Colin-Ramirez et al., 2015 [47]	Patients with chronic HF and NYHA II–III and receiving GDMT/3-day food records with counseling verification	Outpatient/ HFrEF or HFpEF	38	Sodium restriction at 1500 mg/d/ (180 days)	Low sodium intake (2300 mg/d)	NS difference in mortality, NYHA class or QoL
Machado d’ Almeida et al., 2018 [48]	Hospitalized patients with ADHF and LVEF > 50%/Hospital nutrition records and sodium content of meals	Inpatient/ HFpEF	53	Very low sodium restriction at 800 mg/d and fluid restriction/ (7 days)	Standard hospital diet: 4000 mg/d sodium and unlimited fluid intake	30-day mortality or readmission rate: NS
Hummel et al. 2018 [49]	Patients ≥ 65 y with history of hypertension, discharged from hospital with ADHF/3-day food records analyzed using standardized software	Outpatient/ HFrEF or HFpEF	66	Sodium-restricted DASH diet with 1500 mg/d sodium/ (30 days)	Routine dietary advisories + phone calls	30-day mortality or readmission rate: NS
Fabricio et al., 2019 [50]	Patients hospitalized with ADHF/Standardized hospital diet and 24 h urinary sodium	Inpatient/ HFrEF or HFpEF	44	Sodium restriction at 1200 mg/d and fluid restriction/ (7 days)	Normal-sodium diet (2800 mg/d)	30-day readmission rate: NS
Kalogeropoulos et al., 2020 [51]	Patients recently hospitalized for HF, on optimal GDMT, SBP ≥ 100 mm Hg, consuming > 3000 mg Na/day/24 h urinary sodium	Outpatient/ HFrEF	27	Sodium restriction at 1500 mg/d/ (84 days)	Normal-sodium diet (3000 mg/d)	30-day readmission rate: NS
Ivey-Miranda et al., 2021 [52]	Patients with chronic, on optimal GDMT, SBP ≥ 90 mm Hg/24 h urinary sodium with dietary logs	Outpatient/ HFrEF	70	Sodium restriction at 2000 mg/d/ (140 days)	Normal-sodium diet (3000 mg/d)	30-day mortality or readmission rate: NS
Ezekowitz et al., 2022 [53] SODIUM-HF trial	Patients with chronic HF and NYHA functional class II or III/3-day food diaries assessed by blinded dietitians	Inpatient or outpatient/ HFrEF or HFpEF	806	Sodium restriction at 1500 mg/d/ (1 year)	Usual care and routine dietary advisories	30-day mortality or readmission rate: NS

ADHF: Acute decompensated heart failure, CV: Cardiovascular, GDMT: Guideline-directed medical therapy, HF: Heart failure, HFrEF: HF with reduced ejection fraction, HFpEF: HF with preserved ejection fraction, LVEF: Left ventricle ejection fraction, NA: Not applicable, NS: Non significant, NYHA: New York Heart Association, QoL: Quality of life, SBP: Systolic blood pressure.

## Data Availability

No new data were created or analyzed in this study. Data sharing is not applicable to this article.

## Abbreviations

The following abbreviations are used in this manuscript:

ADH	Antidiuretic hormone
ADHF	Acute decompensated heart failure
ANP	Atrial natriuretic peptide
BNP	B-type natriuretic peptide
BP	Blood pressure
CKD	Chronic kidney disease
CO	Cardiac output
CVD	Cardiovascular disease
EF	Ejection fraction
ESC	European Society of Cardiology
NO	Nitric oxide
NYHA	New York Heart Association
HF	Heart failure
HFrEF	HF with reduced ejection fraction
HFpEF	HF with preserved ejection fraction
QoL	Quality of life
RAAS	Renin–angiotensin–aldosterone system
RCT	Randomized controlled trials
SDM	Shared decision-making
SGLT2	Sodium glucose co-transporter 2
SNS	Sympathetic nervous system
